# C5a elevation in convalescents from severe COVID-19 is not associated with early complement activation markers C3bBbP or C4d

**DOI:** 10.3389/fimmu.2022.946522

**Published:** 2022-08-24

**Authors:** Daria Kowalska, Alicja Kuźniewska, Yaiza Senent, Beatriz Tavira, Susana Inogés, Ascensión López-Díaz de Cerio, Ruben Pio, Marcin Okrój, José Ramón Yuste

**Affiliations:** ^1^ Department of Cell Biology and Immunology, Intercollegiate Faculty of Biotechnology, University of Gdańsk and Medical University of Gdańsk, Gdańsk, Poland; ^2^ Program in Solid Tumors, Translational Oncology Group, Cima-University of Navarra and Cancer Center University of Navarra (CCUN), Pamplona, Spain; ^3^ Department of Biochemistry and Genetics, School of Sciences, University of Navarra, Pamplona, Spain; ^4^ Department of Oncology and Hematology, Navarra Institute for Health Research (IdISNA), Pamplona, Spain; ^5^ Department of Pathology, Anatomy and Physiology, School of Medicine, University of Navarra, Pamplona, Spain; ^6^ Department of Immunology and Immunotherapy, Clinica Universidad de Navarra, Pamplona, Spain; ^7^ Area of Cell Therapy and Department of Hematology, Clinica Universidad de Navarra, Pamplona, Spain; ^8^ Program in Respiratory Tract Tumors, Centro de Investigación Biomédica en Red de Cáncer (CIBERONC), Madrid, Spain; ^9^ Department of Internal Medicine, Clinica Universidad de Navarra, Pamplona, Spain; ^10^ Division of Infectious Diseases, Clinica Universidad de Navarra, Pamplona, Spain

**Keywords:** COVID-19, complement system, C5a, anaphylatoxin, clinical risk score

## Abstract

Numerous publications have underlined the link between complement C5a and the clinical course of COVID-19. We previously reported that levels of C5a remain high in the group of severely ill patients up to 90 days after hospital discharge. We have now evaluated which complement pathway fuels the elevated levels of C5a during hospitalization and follow-up. The alternative pathway (AP) activation marker C3bBbP and the soluble fraction of C4d, a footprint of the classical/lectin (CP/LP) pathway, were assessed by immunoenzymatic assay in a total of 188 serial samples from 49 patients infected with SARS-CoV-2. Unlike C5a, neither C3bBbP nor C4d readouts rose proportionally to the severity of the disease. Detailed correlation analyses in hospitalization and follow-up samples collected from patients of different disease severity showed significant positive correlations of AP and CP/LP markers with C5a in certain groups, except for the follow-up samples of the patients who suffered from highly severe COVID-19 and presented the highest C5a readouts. In conclusion, there is not a clear link between persistently high levels of C5a after hospital discharge and markers of upstream complement activation, suggesting the existence of a non-canonical source of C5a in patients with a severe course of COVID-19.

## Introduction

The elevated levels of complement C5a anaphylatoxin in the inflammatory response to COVID-19 (C-19) has been independently described by numerous research groups. A high concentration of C5a was associated with disease severity and predicted the outcome of hospitalized patients (1–6). Our recent study showed that C5a was not only correlated with a clinical risk score and the severity of the disease but its high level persisted up to 90 days after hospital discharge in patients experiencing either a harsh course of C-19 or long-term respiratory symptoms (4). Rapid clearance of C5a from serum is ensured by ubiquitous immune cells equipped with high-affinity C5a receptors (7, 8). Yet, resolving of infection did not result in a rapid decrease of free anaphylatoxin in certain patient groups, suggesting the perseverance of mechanisms leading to *de novo* C5a generation. Therefore, we decided to trace upstream events in the complement cascade that normally precede C5a generation. C5a, a proteolytic fragment of the C5 component, can originate from all three complement activation routes: classical (CP), lectin (LP), and alternative (AP) pathways. These pathways converge at the level of the C3 molecule, which is processed by C3 convertases—enzymatic complexes that produce C3b and C3a fragments. The same (C4b2a) C3 convertase acts in CP and LP, while AP uses its own C3 convertase C3bBbP. A soluble, properdin-stabilized AP convertase complex (C3bBbP) is considered as an AP activation marker (9–11). Analogically, a soluble fraction of the C4d fragment, the end degradation product that origins from the C4b subunit of C4b2a convertase, appears proportionally to the degree of CP/LP activation. Neoepitope-based detection of C4d by ELISA was previously used to mirror prior complement activation (11–14).

## Methods

### Patients

The characteristics of the patients enrolled in the study, clinical criteria, blood collection, and serum preparation as well as quantification of C5a and TCC complement activation markers were described in detail in (4). Patients were divided into different severity groups based on the hospitalization status and the length of the hospitalization period, according to the following criteria: very low severity = no hospitalization, low severity = from 5 to 7 days of hospitalization, medium severity = from 8 to 13 days of hospitalization, high severity = from 14 days of hospitalization, very high severity = patients who deceased. Such classification closely mirrors the clinical risk score (CRS) for C-19 patients as well as the CRS-based probability of developing a critical illness described in detail by Liang et al. in *JAMA Intern Med., 2020* (15) (see **Figure S1**). Briefly, the CRS was validated on 1,590 patients and it is based on 10 parameters considered as independent predictive factors, including chest radiographic abnormality, age, hemoptysis, dyspnea, unconsciousness, cancer history, neutrophil-to-lymphocyte ratio, lactate dehydrogenase concentration, direct bilirubin, and the total number of comorbidities from the set of chronic obstructive pulmonary disease, hypertension, diabetes, coronary heart disease, chronic kidney disease, cancer, cerebral vascular disease, hepatitis B, and immunodeficiency (15). Samples from hospitalization were collected around days 1, 5, 8, and 14. The follow-up samples were collected around 7, 14, or 90 days after hospital discharge, with the median values of 14.5 days (range 7–106), 14 days (6–105), or 17 days (5–97) for low-, medium-, and high-severity groups, respectively. Detailed information for every single serum sample is presented in **Table S1**. The study was approved by the Ethics Committee of Clinica Universidad de Navarra (ref. 2020.090), and all patients signed informed consent.

### Quantification of C4d and C3bBbP

Markers of complement activation are usually measured in EDTA-plasma as the chelation of divalent cations prevents the initiation of complement cascade in the test tube (16). For this reason, serum is not the preferred clinical material for the assessment of complement activation markers. Still, if no plasma samples are available, certain precautions can be made to reduce artifact formation in serum. Accordingly, we performed our measurements in serum samples diluted in buffer containing EDTA that blocks *de novo* formation of complement convertases (9). Additionally, blood samples were processed immediately after collection and obtained serum samples were aliquoted and frozen at -80°C until the determination of complement activation markers. Ninety-six-well Maxisorp plates (Nunc) were coated overnight at room temperature with 10 μg/ml of C-terminal neoepitope-specific anti-C4d antibody [described in (12)], diluted in PBS + sodium azide (0.02%). Afterward, plates were blocked for 1 h at 37°C with 3% fish skin gelatin solution (Sigma) in washing buffer (140 mM NaCl, 20 mM Tris–HCl pH 7.4, 0.2% Tween 20) and incubated with 2.5% of patients’ sera diluted in AG buffer (PBS buffer with 0.02% Tween 20 and 20 mM EDTA) for 1 h at room temperature. Serial dilutions of recombinant C4d preparation (14) in AG buffer with 2.5% normal human serum were used to prepare a standard curve. C4d was detected by mouse anti-human C4d antibodies (Quidel, cat. No. #253) diluted 1:1,500 in PBS with 0.02% Tween 20 followed by the secondary HRP-conjugated goat anti-mouse antibody (Dako) at dilution 1:5,000. Test was developed by the addition of TMB solution (Sigma), and the color reaction was stopped by the addition of 0.5 M H_2_SO_4_. Absorbance at 450 nm was read by a Synergy H1 microplate reader (BioTek).

The assessment of C3bBbP levels was similar to C4d, but 96-well plates were coated overnight at 4°C with mouse monoclonal anti-factor P antibody (#P1, Quidel) (9) 1:1,000 and blocked with 3% BSA in carbonate buffer for 1 h 37°C. Then they were overlaid with 2.5% of patients’ sera in AG buffer and incubated for 1 h at 4°C. A standard curve was made by serial dilutions (from 1:50 to 1:3,200) of zymosan-activated serum attributable to the ICS#2 standard (9) and defined as 1,000 CAU (complement activating units) per 1 ml. For the detection, wells were incubated with 1:1,000 dilution of goat anti-human C3 (Complement Technology) followed by 1:1,000 HRP-conjugated rabbit anti-goat antibodies (Dako).

## Results

**Figure 1:**
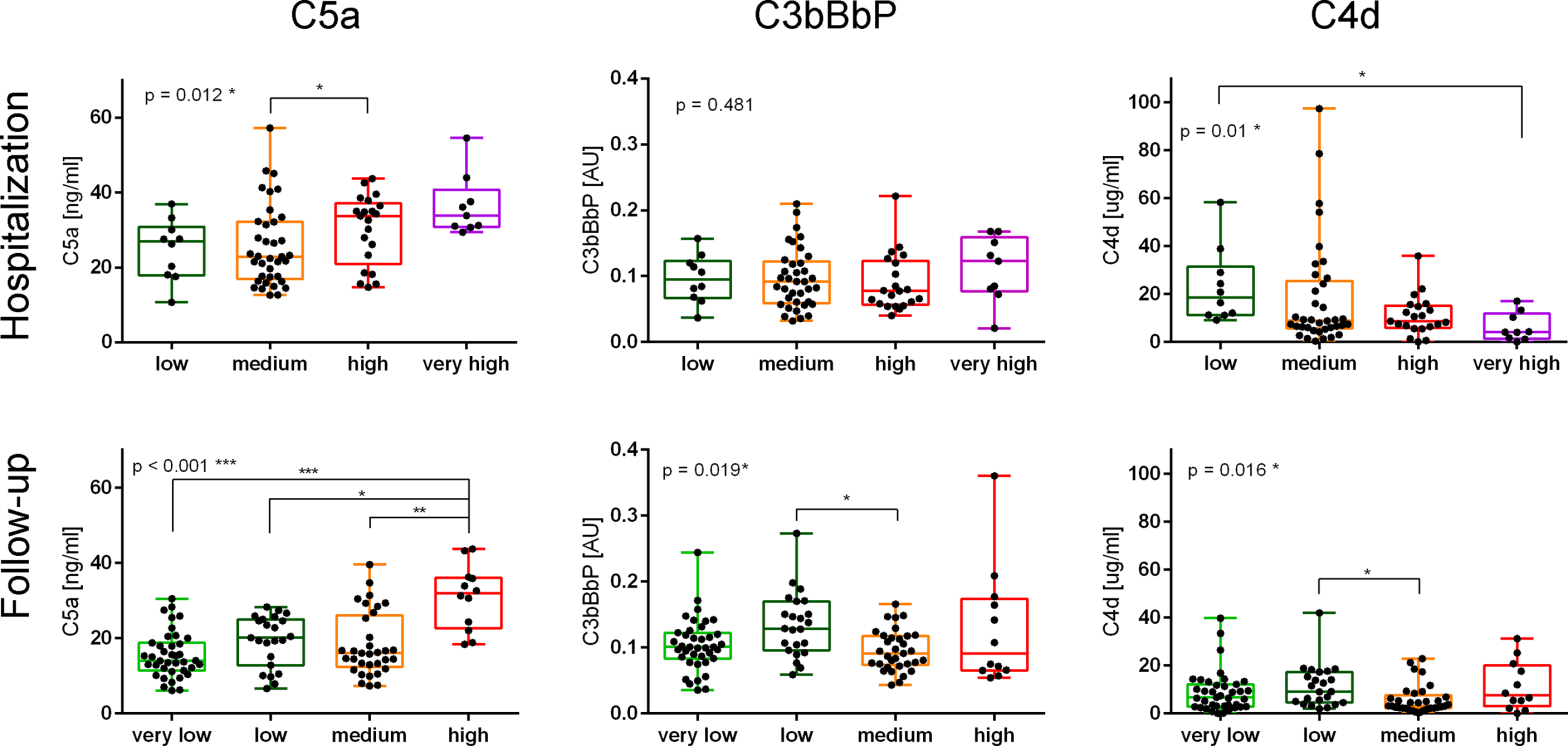
Markers of complement activation in samples collected from patients with different severities of COVID-19 during hospitalization and follow-up. Serum samples collected from COVID-19 patients during the hospitalization and follow-up were divided into groups based on the disease severity. Patients classified as “very low” severity did not need hospitalization, and therefore only follow-up samples were available. Conversely, patients classified to the “very high” severity group died during the hospitalization. The other severity groups were set up according to the number of days up to the hospital discharge: ≤7 for a low-severity group, 8–13 days for a medium-severity group, and ≥14 for a high-severity group. The results of the C5a measurement were originally published in ref. 4; herein, we present the data obtained for each severity group during hospitalization and follow-up. Symbols *, **, and *** indicate p values <0.05, <0.01, and <0.001, respectively, according to the Kruskal–Wallis test with Dunn’s multiple-comparison posttest.

**Figure 2:**
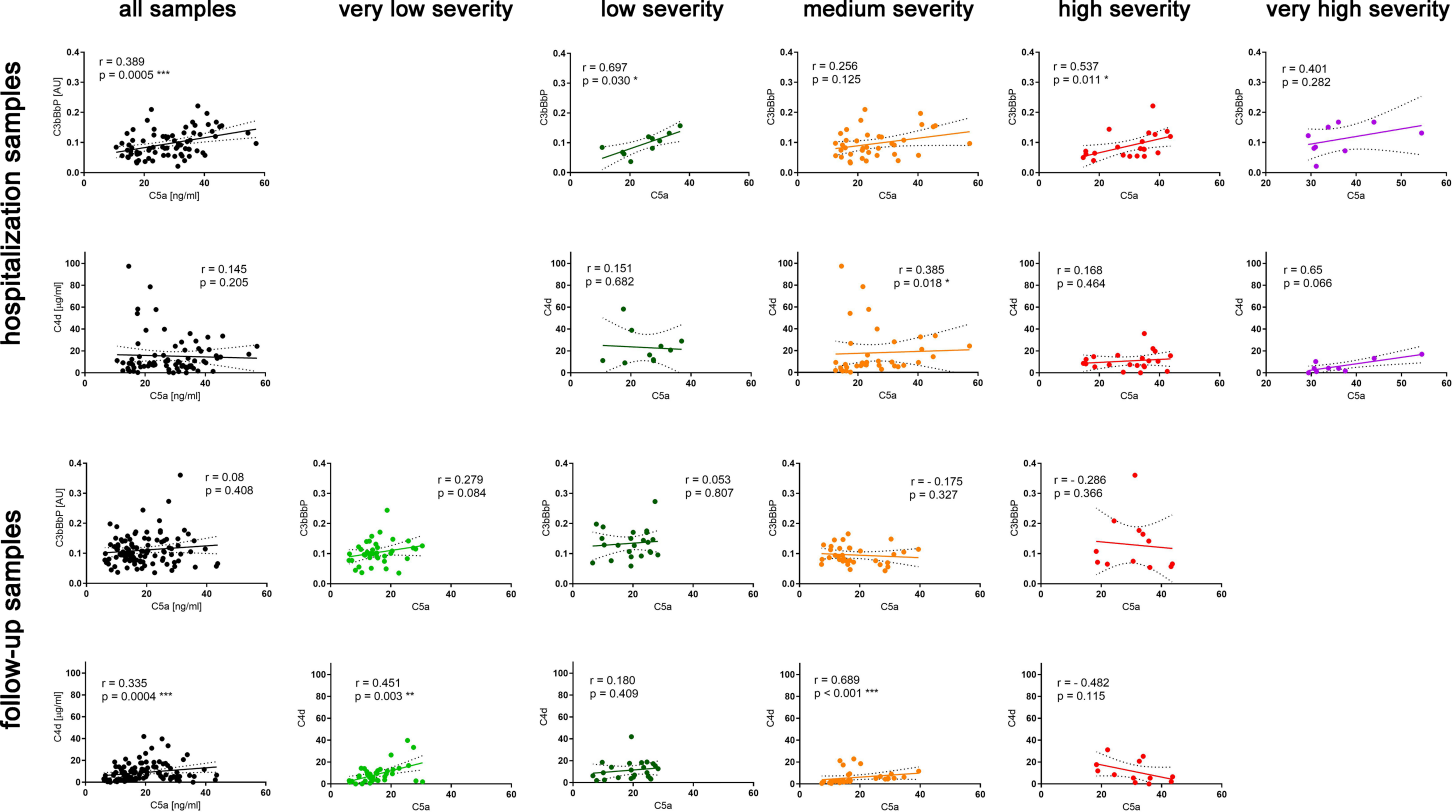
Correlations of C5a with C3bBbP and C4d readouts in hospitalization and follow-up serum samples. Spearman r coefficients and p values were calculated for all hospitalization and follow-up samples and separately for each severity group. The 95% confidence band of the best-fit line is shown. Symbols *, **, and *** denote statistical significance at p level <0.05, <0.01, or < 0.001, respectively.

In contrast to C5a (4), C3bBbP and C4d values did not increase in parallel to disease severity (**Figure 1**) and none of the two markers correlated with the C-19 clinical risk score (**Figure S2**). While analyzing early complement activation markers in all samples collected during hospitalization or the follow-up period (and not taking into account particular severity groups), a significant correlation between C5a and C3bBbP levels was observed in hospitalization samples (Spearman r = 0.389, p < 0.001) (**Figure 2**, top panel) but not in the follow-up samples. On the other hand, a significant correlation between C4d and C5a values was observed in follow-up samples (r = 0.335, p < 0.001) (**Figure 2**, bottom panel) but not in hospitalization samples, indicating that CP/LP may take over the AP in the postinfection course. When the same analyses were performed in particular disease severity groups, the strongest correlation between C5a and C3bBbP (r = 0.697, p < 0.03) was observed in the group of hospitalized patients with low C-19 severity, and the strongest correlation between C5a and C4d was observed in follow-up patients with medium C-19 severity (r = 0.689, p < 0.001). Interestingly, no correlation and inverse interrelationships between C5a:C3bBbP and C5a:C4d readouts were noticed in the follow-up samples of patients with high C-19 severity, who showed the highest levels of C5a among all individuals enrolled in follow-up.

**Figure 3:**
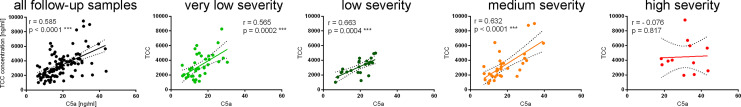
Correlations of C5a and TCC in patients’ serum samples collected during follow-up. The results of the C5a and TCC measurements were originally published in ref. 1; herein, we present the correlation of data obtained for all follow-up samples and for each severity group separately. The 95% confidence band of the best-fit line is shown. Symbols *** denotes statistical significance at p level < 0.001.

The terminal complement complex (TCC) is a marker of complement activation downstream of C5. TCC consists of a soluble fraction of C5b6-9 components released into the fluid phase proportionally to the amount of the membrane attack complex (9). As expected, C5a readouts in all follow-up samples correlated well with TCC (Spearman r = 0.585, p < 0.001) (**Figure 3**). However, when analyzing particular groups of patients, C5a and TCC readouts remained highly correlated in patients with very low- (r = 0.565), low- (r = 0.663), and medium-severity C-19 (r = 0.632), but not in those of high severity (**Figure 3**).

## Discussion

Previously, we determined C5a levels in longitudinally collected serum samples from 49 patients suffering from C-19 with different clinical intensities and concluded that serum C5a levels remain high in severe cases of COVID-19 and are associated with the persistence of respiratory symptoms after hospital discharge (4). An increase in C5a proportional to the severity of the disease has been also reported in other studies using either plasma (1, 2, 6) or serum (3–5). The observation that C5a, normally cleared rapidly from the circulation (7, 8), remains elevated in follow-up samples from COVID-19 convalescents made us investigate the source of this anaphylatoxin after the clearance of infection. The C5a levels in samples collected during hospitalization and follow-up correlated with one of the upstream complement activation markers C3bBbP or C4d. This result is in line with other studies that report elevated AP markers in C-19 patients during hospitalization and direct AP activation by SARS-CoV-2 spike protein (17, 18). Notably, the follow-up samples collected from severely ill patients that contained the highest levels of C5a showed an inverse pattern with a negative Spearman r coefficient (**Figure 2**). Moreover, unlike the samples from other disease severity groups, these samples did not correlate with TCC levels (**Figure 3**), suggesting that the elevated C5a levels that persist in the most severely ill patients are not associated with neither downstream nor upstream complement activation markers.

One of the hypotheses behind elevated C5a during the C-19 course involves the pro-coagulant state in the microvasculature of the lungs and kidneys (19). Notably, a strong correlation between C5a and von Willebrand factor was observed in C-19 patients (3). Regarding the cross talk of complement and the coagulation system, an important observation was made by Huber-Lang et al. in mice genetically lacking the C3 protein. Elimination of this central complement component did not disable the C5 convertase activity, and generation of biologically active C5a was eventually abolished by thrombin inhibitors (20). The studies performed in physiological *ex vivo* conditions revealed that efficient cleavage of human C5 by thrombin occurs only after temporal acidification that changes native C5 conformation (21). Such conditions can be applicable in pathophysiological settings as low pH is characteristic for sites of inflammation (22). Nonetheless, purified human C5 can generate C5a when exposed to thrombin but also FIXa, FXa, FXIa, and plasmin (22, 23); additionally, the FXa-dependent cleavage of C5 in human serum was demonstrated by Amara et al. (23)

Krisinger et al. reported that thrombin processes C5 at the alternate cleavage site (arginine 947), thus leading to a functionally active but structurally different C5b6-9 complex from the one generated by the complement C5 convertases (24). If a portion of C5 originally present in the serum of severe C-19 convalescents was processed in a similar manner, such C5b-9 complex might escape detection by our TCC assay that uses a monoclonal antibody (clone ae11) specific to a conformational neoepitope on C5b-9 (25). This could explain the lack of correlation between C5a and TCC in follow-up samples collected from convalescents who experienced a harsh course of C-19.

Since the critical role of C5a in the pathogenesis of severe C-19 was acknowledged, Annane et al. performed a non-controlled clinical study in patients admitted to the intensive care unit, to whom C5 blocker eculizumab was administered (26). Eculizumab, a monoclonal antibody approved for the treatment of complement-related renal diseases like aHUS and PNH, binds C5 in a way that disables cleavage, thus preventing C5a generation and C5b-driven assembly of the terminal complex (27). Although the administration of eculizumab resulted in a higher survival rate compared to standard care, the median concentrations of C5a did not significantly differ between eculizumab-treated and eculizumab-free patients (28). The reason for the incomplete C5 inhibition remains unknown but may involve either very robust complement activation in severely ill patients or a different mechanism of C5 processing. Altogether with our results showing that patients experiencing a severe course of C-19 did not show a correlation between their high levels of C5a and markers reflecting the upstream stages of the complement cascade, all the abovementioned remarks speak for the necessity of elucidating the origin of C5a that may be associated with adverse effects in C-19 patients. Further experiments are necessary to evaluate a possible contribution of the enzymes of the coagulation cascade, which then might become a target for the therapy of post-COVID patients.

## Data availability statement

The original contributions presented in the study are included in the article/**Supplementary Material**. Further inquiries can be directed to the corresponding author.

## Ethics statement

The studies involving human participants were reviewed and approved by Ethics Committee of Clinica Universidad de Navarra (Ref. 2020.090). The patients/participants provided their written informed consent to participate in this study.

## Author contributions

Conceptualization: YS, BT, RP, MO, and JY; resources: DK and MO; collection of clinical samples, processing, and data management: AL-DC, SI, and JY; experimental work: DK, AK, and MO; data analysis: YS, RP, BT, JY, and MO; writing of the draft of the manuscript: MO and RP. All authors contributed to the article and approved the submitted version.

## Funding

This work was supported by the National Science Centre Poland (2019/35/B/NZ6/02450), Foundation for Applied Medical Research (FIMA), Fundación MTorres, Fundación Ramón Areces, Fondo de Investigación Sanitaria-Fondo Europeo de Desarrollo Regional “Una manera de hacer Europa” (PI20/00419), Departamento de Salud de Gobierno de Navarra (0011-3638-2020-000004), and Departamento de Desarrollo Económico y Empresarial del Gobierno de Navarra (AGATA and DESCARThES projects). UGrants Start 2022 no. 1220/96/2022 from the University of Gdańsk.

## Conflict of interest

The authors declare that the research was conducted in the absence of any commercial or financial relationships that could be construed as a potential conflict of interest.

## Publisher’s note

All claims expressed in this article are solely those of the authors and do not necessarily represent those of their affiliated organizations, or those of the publisher, the editors and the reviewers. Any product that may be evaluated in this article, or claim that may be made by its manufacturer, is not guaranteed or endorsed by the publisher.

## Abbreviations

C-19, COVID-19; AP, alternative complement pathway; CP, classical complement pathway; CRS, clinical risk score; ELISA, enzyme-linked immunosorbent assay; LP, lectin complement pathway.
