# Supplementary material for: C5a elevation in convalescents from severe COVID-19 is not associated with early complement activation markers C3bBbP or C4d
Source: Front Immunol. 2022 Aug 24;13:946522. doi: 10.3389/fimmu.2022.946522 (PMC9448977; doi:10.3389/fimmu.2022.946522)
Supplement: Supplementary file 1 [file Table_1.docx]

**Supplementary Table 1 Detailed information on serum samples collection**

| **Patient** | **Hospitalization** | **Disease severity** | **Days from diagnosis** | **Days from hospital discharge** | **Sample type** |
| --- | --- | --- | --- | --- | --- |
| Patient 1 | No | very low | 16 |  | F14 |
| Patient 1 | No | very low | 107 |  | F90 |
| Patient 2 | No | very low | 25 |  | F14 |
| Patient 2 | No | very low | 111 |  | F90 |
| Patient 3 | No | very low | 32 |  | F14 |
| Patient 3 | No | very low | 123 |  | F90 |
| Patient 4 | No | very low | 17 |  | F14 |
| Patient 4 | No | very low | 108 |  | F90 |
| Patient 5 | No | very low | 14 |  | F14 |
| Patient 5 | No | very low | 110 |  | F90 |
| Patient 6 | No | very low | 13 |  | F14 |
| Patient 6 | No | very low | 117 |  | F90 |
| Patient 7 | No | very low | 19 |  | F14 |
| Patient 7 | No | very low | 113 |  | F90 |
| Patient 8 | No | very low | 10 |  | F7 |
| Patient 8 | No | very low | 20 |  | F14 |
| Patient 8 | No | very low | 99 |  | F90 |
| Patient 9 | No | very low | 17 |  | F14 |
| Patient 9 | No | very low | 123 |  | F90 |
| Patient 10 | No | very low | 16 |  | F14 |
| Patient 11 | No | very low | 12 |  | F7 |
| Patient 11 | No | very low | 18 |  | F14 |
| Patient 11 | No | very low | 106 |  | F90 |
| Patient 12 | No | very low | 19 |  | F7 |
| Patient 12 | No | very low | 28 |  | F14 |
| Patient 12 | No | very low | 122 |  | F90 |
| Patient 13 | No | very low | 15 |  | F7 |
| Patient 13 | No | very low | 22 |  | F14 |
| Patient 13 | No | very low | 112 |  | F90 |
| Patient 14 | No | very low | 15 |  | F14 |
| Patient 14 | No | very low | 89 |  | F90 |
| Patient 15 | No | very low | 17 |  | F14 |
| Patient 15 | No | very low | 95 |  | F90 |
| Patient 16 | No | very low | 9 |  | F7 |
| Patient 16 | No | very low | 16 |  | F14 |
| Patient 16 | No | very low | 107 |  | F90 |
| Patient 17 | No | very low | 23 |  | F7 |
| Patient 17 | No | very low | 33 |  | F14 |
| Patient 17 | No | very low | 106 |  | F90 |
| Patient 18 | Yes | low | 28 | 9 | F7 |
| Patient 18 | Yes | low | 37 | 18 | F14 |
| Patient 18 | Yes | low | 104 | 85 | F90 |
| Patient 19 | Yes | low | 24 | 9 | F7 |
| Patient 19 | Yes | low | 29 | 14 | F14 |
| Patient 19 | Yes | low | 120 | 105 | F90 |
| Patient 20 | Yes | low | 20 | 9 | F7 |
| Patient 20 | Yes | low | 26 | 15 | F14 |
| Patient 20 | Yes | low | 103 | 92 | F90 |
| Patient 21 | Yes | low | 23 | 9 | F7 |
| Patient 21 | Yes | low | 30 | 16 | F14 |
| Patient 21 | Yes | low | 102 | 88 | F90 |
| Patient 22 | Yes | low | 22 | 7 | F7 |
| Patient 22 | Yes | low | 29 | 14 | F14 |
| Patient 22 | Yes | low | 121 | 106 | F90 |
| Patient 24 | Yes | low | 18 | 8 | F7 |
| Patient 24 | Yes | low | 24 | 14 | F14 |
| Patient 24 | Yes | low | 82 | 72 | F90 |
| Patient 25 | Yes | low | 22 | 7 | F7 |
| Patient 25 | Yes | low | 29 | 14 | F14 |
| Patient 25 | Yes | low | 112 | 97 | F90 |
| Patient 26 | Yes | low | 40 | 8 | F7 |
| Patient 26 | Yes | low | 47 | 15 | F14 |
| Patient 26 | Yes | low | 105 | 73 | F90 |
| Patient 27 | Yes | medium | 21 | 7 | F7 |
| Patient 27 | Yes | medium | 28 | 14 | F14 |
| Patient 27 | Yes | medium | 119 | 105 | F90 |
| Patient 28 | Yes | medium | 26 | 7 | F7 |
| Patient 28 | Yes | medium | 32 | 13 | F14 |
| Patient 28 | Yes | medium | 124 | 105 | F90 |
| Patient 29 | Yes | medium | 22 | 7 | F7 |
| Patient 29 | Yes | medium | 90 | 75 | F90 |
| Patient 30 | Yes | medium | 27 | 10 | F7 |
| Patient 30 | Yes | medium | 33 | 16 | F14 |
| Patient 30 | Yes | medium | 114 | 97 | F90 |
| Patient 31 | Yes | medium | 23 | 7 | F7 |
| Patient 31 | Yes | medium | 29 | 13 | F14 |
| Patient 31 | Yes | medium | 105 | 89 | F90 |
| Patient 32 | Yes | medium | 18 | 7 | F7 |
| Patient 32 | Yes | medium | 25 | 14 | F14 |
| Patient 32 | Yes | medium | 110 | 99 | F90 |
| Patient 33 | Yes | medium | 17 | 7 | F7 |
| Patient 33 | Yes | medium | 24 | 14 | F14 |
| Patient 33 | Yes | medium | 91 | 81 | F90 |
| Patient 34 | Yes | medium | 30 | 7 | F7 |
| Patient 34 | Yes | medium | 36 | 13 | F14 |
| Patient 34 | Yes | medium | 121 | 98 | F90 |
| Patient 35 | Yes | medium | 26 | 7 | F7 |
| Patient 35 | Yes | medium | 111 | 92 | F90 |
| Patient 36 | Yes | medium | 25 | 7 | F7 |
| Patient 36 | Yes | medium | 33 | 15 | F14 |
| Patient 36 | Yes | medium | 113 | 95 | F90 |
| Patient 37 | Yes | medium | 22 | 10 | F7 |
| Patient 37 | Yes | medium | 30 | 18 | F14 |
| Patient 37 | Yes | medium | 107 | 95 | F90 |
| Patient 38 | Yes | medium | 25 | 6 | F7 |
| Patient 38 | Yes | medium | 33 | 14 | F14 |
| Patient 40 | Yes | high | 28 | 7 | F7 |
| Patient 40 | Yes | high | 35 | 14 | F14 |
| Patient 40 | Yes | high | 118 | 97 | F90 |
| Patient 41 | Yes | high | 37 | 20 | F14 |
| Patient 41 | Yes | high | 114 | 97 | F90 |
| Patient 42 | Yes | high | 36 | 10 | F7 |
| Patient 42 | Yes | high | 108 | 82 | F90 |
| Patient 43 | Yes | high | 20 | 5 | F7 |
| Patient 43 | Yes | high | 28 | 13 | F14 |
| Patient 43 | Yes | high | 85 | 70 | F90 |
| Patient 45 | Yes | high | 51 | 12 | F14 |
| Patient 45 | Yes | high | 108 | 69 | F90 |
