# Supplementary material for: C5a elevation in convalescents from severe COVID-19 is not associated with early complement activation markers C3bBbP or C4d
Source: Front Immunol. 2022 Aug 24;13:946522. doi: 10.3389/fimmu.2022.946522 (PMC9448977; doi:10.3389/fimmu.2022.946522)
Supplement: Supplementary file 2 [file DataSheet_1.docx]

Fig. S1

Classification of severity groups based on the length of hospitalization period vs. CRS-based probability of developing a critical illness (according to *Liang et al.* in *JAMA Intern Med., 2020* ^15^)

C-19 severity groups were established based on the length of the hospitalization period, according to the following criteria: very low severity = no hospitalization, low severity = from 5 to 7 days of hospitalization, medium severity = from 8 to 13 days of hospitalization, high severity = from 14 days of hospitalization, very high severity = patients who deceased. Sidak’s multiple comparison test revealed statistically significant differences between the hospitalized patients classified into consecutive severity groups. *, **, and *** denote statistical significance at the p levels of <0.05, <0.01, and <0.001, respectively.
