# Supplementary material for: C5a elevation in convalescents from severe COVID-19 is not associated with early complement activation markers C3bBbP or C4d
Source: Front Immunol. 2022 Aug 24;13:946522. doi: 10.3389/fimmu.2022.946522 (PMC9448977; doi:10.3389/fimmu.2022.946522)

Fig. S2 Correlations between complement activation markers in the first samples collected from hospitalized patients and COVID-19 clinical risk score.


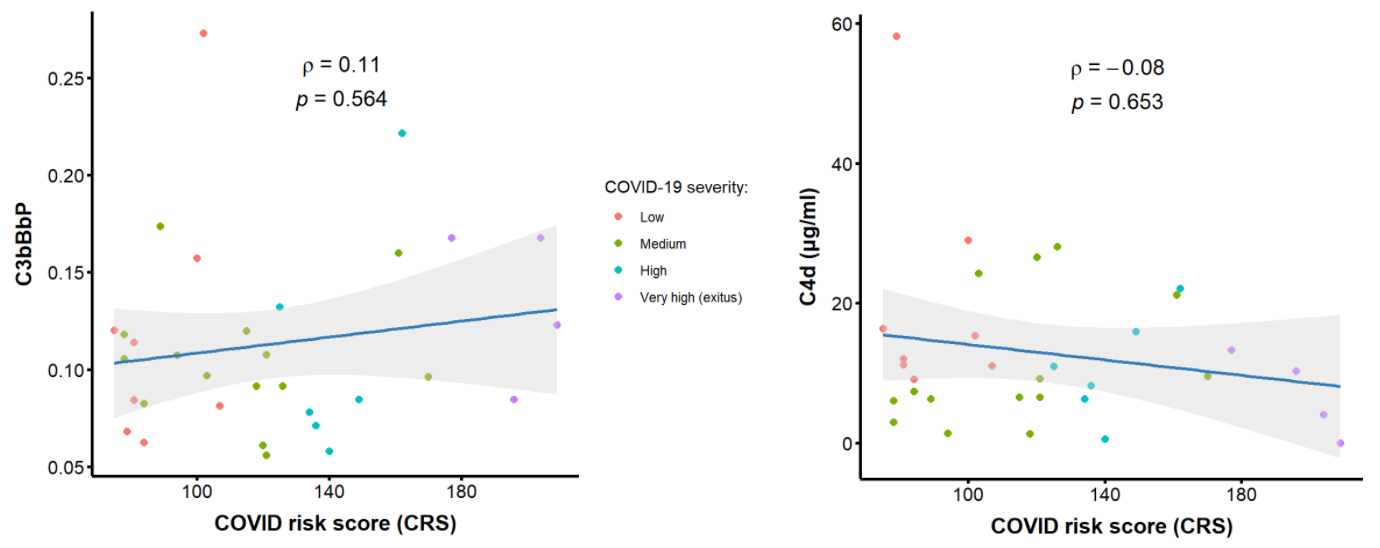

Supplement: Supplementary file 3 [file DataSheet_2.docx]
